# Roles of the Dynamic Tumor Immune Microenvironment in the Individualized Treatment of Advanced Clear Cell Renal Cell Carcinoma

**DOI:** 10.3389/fimmu.2021.653358

**Published:** 2021-03-04

**Authors:** Enyu Lin, Xuechao Liu, Yanjun Liu, Zedan Zhang, Lu Xie, Kaiwen Tian, Jiumin Liu, Yuming Yu

**Affiliations:** ^1^Department of Urology, Guangdong Provincial People's Hospital, Guangdong Academy of Medical Sciences, Guangzhou, China; ^2^Shantou University Medical College, Shantou, China; ^3^Department of Gastrointestinal Surgery, Affiliated Hospital of Qingdao University, Qingdao, China; ^4^Department of Immunology, School of Basic Medical Science, Southern Medical University, Guangzhou, China

**Keywords:** clear cell renal cell carcinoma, tumor immune microenvironment, immunotherapy, targeted therapy, genomic characteristics

## Abstract

Immune checkpoint inhibitors (ICIs) are currently a first-line treatment option for clear cell renal cell carcinoma (ccRCC). However, recent clinical studies have shown that a large number of patients do not respond to ICIs. Moreover, only a few patients achieve a stable and durable response even with combination therapy based on ICIs. Available studies have concluded that the response to immunotherapy and targeted therapy in patients with ccRCC is affected by the tumor immune microenvironment (TIME), which can be manipulated by targeted therapy and tumor genomic characteristics. Therefore, an in-depth understanding of the dynamic nature of the TIME is important for improving the efficacy of immunotherapy or combination therapy in patients with advanced ccRCC. Here, we explore the possible mechanisms by which the TIME affects the efficacy of immunotherapy and targeted therapy, as well as the factors that drive dynamic changes in the TIME in ccRCC, including the immunomodulatory effect of targeted therapy and genomic changes. We also describe the progress on novel therapeutic modalities for advanced ccRCC based on the TIME. Overall, this review provides valuable information on the optimization of combination therapy and development of individualized therapy for advanced ccRCC.

## Introduction

As one of the most common malignancies of the genitourinary system, renal cell carcinoma (RCC) affects ~400,000 people worldwide each year, resulting in ~175,000 deaths (1). The most common histological type of RCC is clear cell RCC (ccRCC), accounting for about 80% of all cases (2, 3). Unlike other urinary tumors, ccRCC is insensitive to chemotherapy. In 2005, sorafenib, the first anti-angiogenic drug, was approved by the US Food & Drug Administration (FDA) to treat patients with advanced RCC. Although anti-angiogenic agents have been continuously optimized over the last decade to improve response rates and safety, many patients will still develop primary or acquired resistance. Therefore, the use of immunotherapy has been explored for metastatic renal cell carcinoma (mRCC) patients who have developed resistance to anti-angiogenic drugs, including immune checkpoint inhibitors (ICIs).

ICIs can achieve excellent therapeutic outcomes in several cancer types, including ccRCC (4–6). However, only a small proportion of patients responded to ICI monotherapy, and the improvement in overall survival (OS) as a result of ICIs is largely attributed to the long-term survival in a minority of patients (7). Additionally, several studies have shown that vascular endothelial growth factor (VEGF) inhibitors promoted T cell infiltration and reversed the inhibitory effect on antigen-presenting cells (APCs) (8–10). These findings provided the theoretical foundations for combination therapy involving anti-angiogenic drugs and ICIs. However, not all patients benefit from the current combination regimens. Therefore, as more therapeutic options for mRCC become available, individualized treatment (i.e., using different combinations and sequences of treatments for different patients) will be critical for optimizing clinical outcomes.

Of note, the therapeutic effects of anti-angiogenic drugs and ICIs are influenced by the tumor immune microenvironment (TIME). Several studies suggest that high levels of tumor-associated macrophages (TAMs), interleukin-8 (IL-8), and IL-6 in the TIME are related to the poor therapeutic effect of anti-angiogenic drugs (11–18). Moreover, high levels of myeloid-derived suppressor cells (MDSCs) and overexpression of immune checkpoints (e.g., LAG-3 and Tim-3) can lead to resistance to ICIs (14–18). Furthermore, a dynamic change in the TIME was observed during treatment with VEGF/VEGFR inhibitors and mammalian target of rapamycin (mTOR) inhibitors (19, 20). Indeed, relatively unique genomic alterations in ccRCC (e.g., VHL mutations and PBRM1 mutations) may also impact the TIME (21, 22). Therefore, ccRCC with different genomic signatures may respond differently to various treatments. The abovementioned results may partially explain the inconsistent efficacy of ICIs or combination therapy for ccRCC.

Herein, we briefly describe the characteristics of the TIME in ccRCC and present detailed analysis of the mechanism by which the TIME influences immunotherapy response. In particular, we discuss the bidirectional relationship between targeted therapy and TIME, and the tumor genomic signature that manipulates the TIME. Finally, we present the progress in the treatment of advanced ccRCC based on the TIME. Overall, this review provides some insights into the optimization of combination therapy and development of individual treatment options for patients with advanced ccRCC.

### The Relatively Unique TIME in ccRCC

In contrast to other tumor types, the TIME in ccRCC is characterized by a high level of immune cell infiltration and a high degree of angiogenesis. Several pan-cancer analyses have shown that ccRCC have prominent inflammatory profiles, which is one of the tumor types with the highest degree of T-cell infiltration (23–25). Chevrier and colleagues showed the major immune cell subsets in ccRCC were T cells (22 different phenotypes) and TAMs (with 17 phenotypes), accounting for ~51 and 31% of immune cells, respectively (26).

High numbers of CD8^+^ tumor-infiltrating T lymphocytes (TILs) typically correlate with a favorable prognosis in most tumors (including prostatic adenocarcinoma, bladder cancer and breast cancer, etc.), except ccRCC (27). Indeed, CD8^+^ TILs in ccRCC are characterized by exhaustion and functional deficiency rather than defective recruitment, and express high levels of immune checkpoint molecules and low levels of Ki-67, which fail to efficiently activate anti-tumor immune responses (26, 28–31). TAMs, MDSCs, and regulatory T cells (Tregs) are the main immunosuppressive cells in the TIME. In ccRCC, the TAMs are mostly similar to CD163^+^ and CD206^+^ M2 macrophages, which have immunosuppressive actions (32). Meanwhile, MDSCs are generated in the bone marrow under pathological conditions such as tumorigenesis (33), and migrate to tumor tissues or peripheral lymphoid organs mainly under the influence of various chemokines secreted by the tumor cells (34). MDSCs are primarily divided into mononuclear MDSCs (M-MDSCs) and polymorphonuclear MDSCs (PMN-MDSCs), with PMN-MDSCs the predominant type in ccRCC (34). Many studies have shown that M2-like TAMs, MDSCs, and Tregs are associated with poor prognosis in ccRCC (35–37).

Tertiary lymphoid structures (TLS) in the TIME are sites of adaptive immune activation, where dendritic cells (DCs) present local cancer antigens to T cells and induce B cell-mediated humoral immunity and differentiation of effector T (Teff) cells (38, 39). Increasingly, researchers have found that a high density of TLS is related to good prognosis in several cancers, including ccRCC (40–42). However, the density of TLS in ccRCC is lower than in other tumors including non-small cell lung cancer (NSCLC), melanoma, and prostate cancer, in both primary and metastatic cases, suggesting that ccRCC cells may impede the formation of TLS (40, 42, 43).

The reduced TLS formation in ccRCC may be due to the influence of the tumor on DCs. DCs have a great degree of functional plasticity, and different microenvironmental signals can determine the functional phenotypes of DCs by affecting their differentiation, maturation, activation, and polarization (44). DCs can be roughly divided into two categories in ccRCC: TLS-DCs, which are characterized as HLA-DR^hi^ CD83^+^ DC-LAMP^+^, and non-TLS-DCs (NTLS-DCs) in the tumor core, characterized as CD209^+^ CD83^−^ (45). Further studies have shown that these two DC subsets have opposite effects on the clinical outcomes of patients with ccRCC, namely, a high density of TLS-DCs and NTLS-DCs correspond to favorable and poor clinical outcomes, respectively (40, 45). Similarly, Figel et al. found that DCs were dominated by CD209^+^ NTLS-DCs in RCC, while CD83^+^ DC-LAMP^+^ TLS-DCs were rare, which indirectly confirms the low density of TLS in ccRCC (46).

In summary, the majority of ccRCC are inflammatory neoplasia showing a high degree of infiltration of exhausted CD8^+^ TILs, which is a prerequisite for the response to ICIs (47). However, immunosuppression from M2-like TAMs, Tregs, MDSCs, and NTLS-DCs in the TIME may also lead to an insensitivity of ccRCC toward immunotherapy, which makes the tumor microenvironment in a proportion of ccRCC patients have immunosuppressive properties. Additionally, the permeability of abnormal neovascularization in ccRCC limits the Teff cell infiltration, which promotes the formation of an immune-silenced microenvironment (8, 48, 49). Indeed, a multi-omics analysis by Clark et al. found that the TIME of ccRCC can be classified into different subtypes, namely the immunoinflammatory subtype with infiltration of CD8^+^ T cells that have high expression of immune checkpoint molecules, the immunosuppressive subtype with predominant infiltration of suppressor cells such as TAMs, and the immune-silenced subtype with active angiogenesis and the lack of immune cell infiltration (50). Different TIME subtypes of ccRCC have different prognoses and may also have different degrees of sensitivity to systemic therapy (50). Several studies have found that ccRCC which responded better to anti-VEGF treatment showed lower levels of immune checkpoint molecules, similar to the immune-silenced subtype of ccRCC described above (51, 52). Thus, the high level of immune infiltration and angiogenic features together build a relatively unique and dynamic TIME in ccRCC, making it an ideal target for precision-targeted immunotherapy or combination therapy.

### Potential Mechanisms Affecting the Efficacy of Immunotherapy in the TIME in ccRCC

Immunotherapy that have been approved and recommended for advanced ccRCC currently includes cytokines and ICIs. In the 1990s, cytokines such as interferon-α (IFN-α) and interleukin-2 (IL-2) that non-specifically activate the anti-tumor immune response began to be used to treat metastatic ccRCC (53). However, high doses of IL-2 can result in capillary leak syndrome and cause multiple organ damage (53). As immune checkpoints are important components in maintaining the immunosuppressive tumor microenvironment, they are an ideal target for immunotherapy. Nowadays, the PD-1/PD-L1 and CTLA-4/CD28 pathways have been extensively studied, and multiple ICIs targeting these pathways have been approved for systemic therapy of advanced ccRCC. Nevertheless, there are still a large proportion of patients have tumor progression after receiving ICIs (5, 54). As opposed to targeted therapies that directly affect tumor cell survival, the anti-tumor effects of immunotherapy are based on the TIME. Reversing the immunosuppressive nature of the TIME and stimulating tumor-infiltrating NK cells and cytotoxic T lymphocytes (CTLs) are key steps in immunotherapy. Thus, the TIME may be a critical factor affecting immunotherapeutic response, particularly the resistance to immunotherapy. This section discusses the mechanism of resistance to cytokines and ICIs in ccRCC from the perspective of the TIME.

### Cytokines

**Figure 1:**
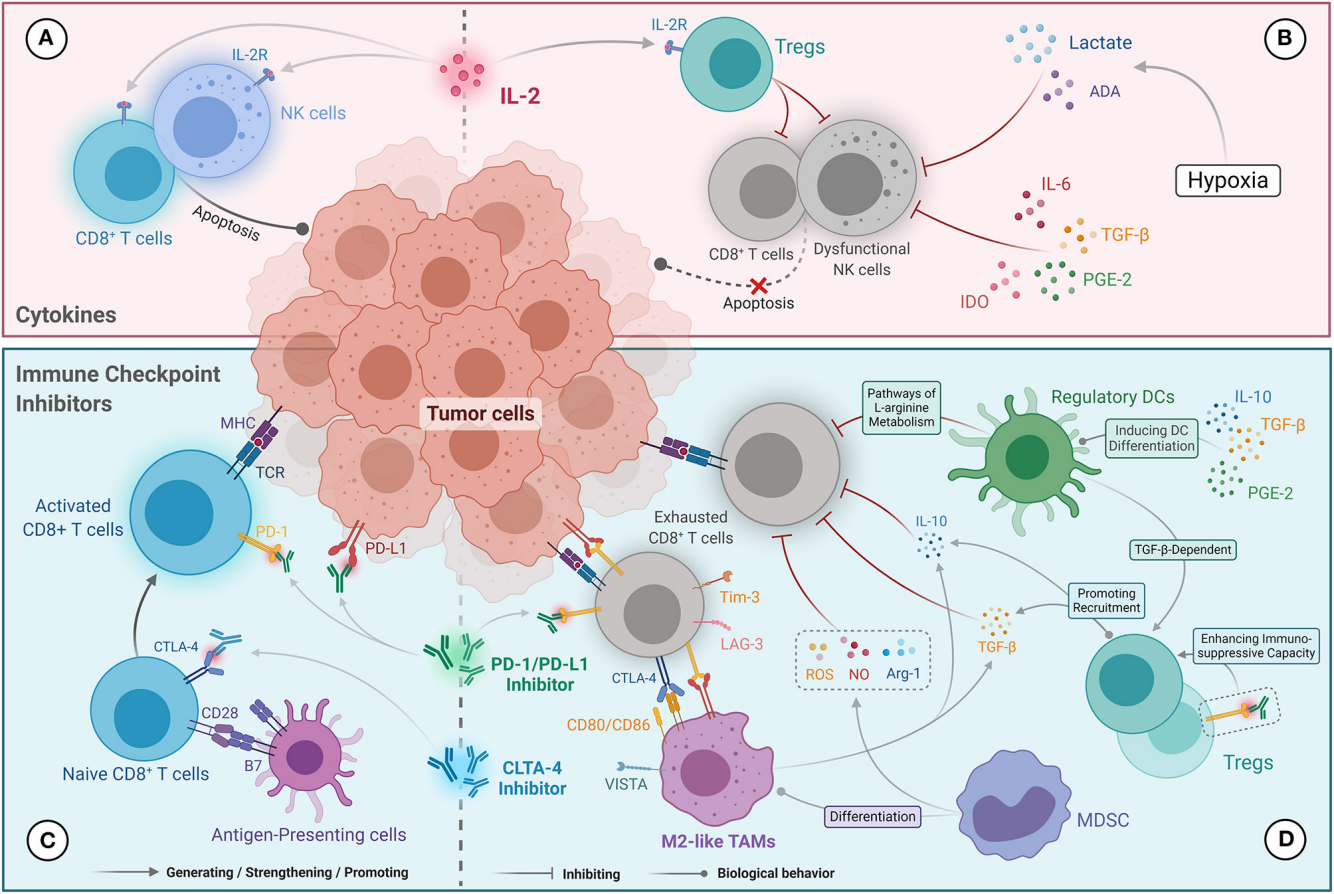
Potential mechanisms influencing immunotherapy response in the TIME in ccRCC. **(A)** The anti-tumor activity of cytokine (IL-2) therapies was primarily mediated by driving the proliferation and activation of NK cells and CD8^+^ TILs. **(B)** Resistance to cytokine (IL-2) therapies was correlated with the amplification of Tregs level mediated by IL-2 and NK cell dysfunction. IL-6, TGF-β, PGE2, and IDO, as well as lactate and adenosine generated under hypoxic conditions, inhibit the cytotoxic effects of NK cells. **(C)** The anti-tumor mechanisms of immune checkpoint inhibitors that contribute to the reactivation of CD8^+^ T cells were mediated by blocking the PD-1/PD-L1 and CTLA-4/CD28 pathways. **(D)** Resistance to immune checkpoint inhibitors is mainly mediated by CD8^+^ TILs anergy, which abundantly express immunosuppressive molecules (e.g., PD-1, CTLA-4, Tim-3, and LAG-3). Tregs and M2-like TAMs secrete IL-10 and TGF-β, which inhibit the cytotoxicity of CD8^+^ TILs and recruit Tregs. Tregs present a stronger immunosuppressive capacity under the action of PD-1 inhibitors. Ligands expressed on M2-like TAMs (including PD-L1/L2, CD80/86, and VISTA) can also promote exhaustion of CD8^+^ TILs. NO, ROS, and Arg-1 produced by MDSCs inhibit the anti-tumor immune function of CD8^+^ TILs, and promote differentiation into M2-like TAMs. Regulatory DCs can also inhibit CD8^+^ T-cell function via the L-arginine metabolic pathway and promote Tregs proliferation.

IL-2 exerts its anti-tumor effect mainly by driving the proliferation and activation of NK cells and CD8^+^ TILs (Figure 1A) (55). However, low-dose IL-2 can induce the preferential amplification of CD4^+^ Foxp3^+^ Tregs, which mainly manifested as a reduction in autoimmune response (Figure 1B) (56, 57). Although high-dose IL-2 has certain ability to activate the anti-tumor immune response, the consequent severe adverse effect and high levels of Tregs significantly limit its clinical benefit. Additional studies have demonstrated that low levels of CD57^+^ NK cells were found to be an independent immune risk factor affecting the prognosis of mRCC patients treated with IL-2, indicating that activated NK cells may be critical for IL-2 to exert its anti-tumor activity (58). Immunosuppressive factors in the tumor microenvironment, including IL-6, TGF-β, PGE2, and indoleamine 2,3-dioxygenase (IDO), can block NK cell activation (59, 60). Moreover, under hypoxic conditions, metabolites in the tumor microenvironment, such as lactate and adenosine, can attenuate the cytotoxic effects of NK cells (Figure 1B) (61, 62). Studies on immunometabolism also revealed that the excessive consumption of glucose and amino acids caused by tumor cell proliferation impairs NK cell proliferation and IFN-γ secretion (60, 63, 64). Prinz et al. verified that tumor-infiltrating NK cells show phenotypic alterations and dysfunction in RCC (primarily poor degranulation activity) compared with those in normal and para-cancerous tissues (65). Based on the above findings, we speculate that the dual immunomodulatory effects of IL-2 and dysfunctional NK cells influenced by the immunosuppressive tumor microenvironment contribute to the insensitivity of ccRCC to IL-2 treatment. Therefore, improving the therapeutic targeting of IL-2 and the activity of NK cells may be effective ways to optimize cytokine therapies.

### Immune Checkpoint Inhibitors

Reversing the exhaustion of CD8^+^ TILs is a key step in the anti-tumor effects of ICIs (Figure 1C) (66). O'Donnell et al. found that exhausted CD8^+^ TILs that only mildly express PD-1 could be reactivated by PD-1/PD-L1 blockade, while over-exhausted CD8^+^ TILs were unresponsive to PD-1/PD-L1 inhibitors (67). The reasons for this phenomenon can be summarized as follows: first, severely exhausted CD8^+^ TILs overexpress PD-1, and thus PD-1/PD-L1 inhibitors cannot completely block the PD-1/PD-L1 signaling pathway to reactivate the T cells. Second, severely exhausted CD8^+^ TILs overexpress other immune checkpoints such as CTLA-4, LAG-3, and Tim-3 (Figure 1D) (67). LAG-3 on CD4^+^ T cells can bind to major histocompatibility complex class II (MHC-II) molecules with a higher affinity than CD4, and directly block T cell receptor signaling, resulting in T cell dysfunction (16). Additionally, LAG-3 on CD8^+^ TILs inhibits the secretion of IFN-γ by binding to two other ligands, galectin-3 and liver sinusoidal endothelial cell lectin (68). Moreover, the binding of Tim-3, a type I transmembrane protein, with a galectin-9 molecule from MDSCs induces dysregulation and apoptosis of CD8^+^ TILs (18). Therefore, in the presence of other upregulated immune checkpoints, only blocking the PD-1/PD-L1 pathway would not reverse T-cell exhaustion.

The current evidence also indicates that severely exhausted Teff cells cannot fully restore effector function even under the influence of ICIs, but can promote resistance to ICIs. An animal model study confirmed that ICIs could rejuvenate T cells that express relatively low levels of PD-1, while relatively high levels of PD-1 were associated with severe T-cell exhaustion and a poor response to ICIs (69). In the phase III JAVELIN renal 101 trial, increased numbers of CD8^+^ TILs in mRCC were associated with improved PFS in the avelumab plus axitinib arm and worse PFS in sunitinib arm (70). Likewise, the phase II IMmotion150 and phase III IMmotion151 trials showed that atezolizumab plus bevacizumab improved PFS compared with sunitinib in mRCC with a high level of Teff cells (14, 71). The findings of these three clinical trials confirm the view that Teff cells play an important role in the therapeutic response to ICIs in mRCC.

In contrast, other studies found no significant correlation between PD-1 levels in mRCC and the benefit of ICIs alone or as a combination therapy, which was not observed in NSCLC and melanoma (14, 54, 70–76). We speculate that severely exhausted CD8^+^ TILs that overexpress PD-1 exist in mRCC, which is in line with the observation that a high infiltration of CD8^+^ TILs is related to poor prognosis in ccRCC. This severe exhaustion of CD8^+^ TILs is likely responsible for resistance to ICIs in mRCC, and also explains why PD-1 status cannot be used alone as a predictor of response to ICI therapy.

Tregs can block the function of T cells and APCs by producing IL-10 and TGF-β to mediate immunosuppression (Figure 1D) (77, 78). Surprisingly, Tregs are activated and proliferate in the presence of PD-1 inhibitors, which confers a poor prognosis (79). In addition, an animal model study found that exhaustion of Tregs could improve the therapeutic response of ICIs (80). Thus, the immunosuppressive properties of Tregs may also contribute to drug resistance or progression in patients treated with PD-1/PD-L1 inhibitors.

IL-10 and TGF-β secreted by M2-like TAMs can also recruit Tregs and directly inhibit the function of CD8^+^ TILs (Figure 1D) (81). M2-like TAMs can also induce T-cell exhaustion by expressing PD-1 ligands (PD-L1 and PD-L2), CTLA-4 ligands (CD80 and CD86), and VISTA (a potent negative regulator of T cell function) (Figure 1D) (81). A recent retrospective study confirmed that high infiltration of M2-like TAMs was associated with poor OS in mRCC patients treated with ICIs (82). Moreover, several animal model studies have found that targeting M2-like TAMs can improve the response to ICIs in various tumors, including pancreatic cancer, colon cancer, breast cancer, and glioblastoma (83). Collectively, M2-like TAMs counteract the anti-tumor effects of ICIs by expressing and secreting immunosuppressive molecules, and participating in immune escape.

MDSCs in ccRCC can significantly inhibit the T cell-specific immune response by producing large amounts of nitric oxide (NO), reactive oxygen species (ROS), and arginase-1 (Arg-1) (Figure 1D) (84). Additionally, MDSCs can also differentiate into M2-like TAMs to mediate immunosuppression (Figure 1D) (85). A significant correlation has been found between high levels of MDSCs and poor treatment response to ICIs in melanoma and prostate cancer (86). Furthermore, the IMmotion150 trial showed that in mRCC patients with a high level of myeloid cells, the combination of atezolizumab and bevacizumab or bevacizumab monotherapy was superior to atezolizumab monotherapy (14). These findings indicate that myeloid cells could lead to the development of resistance to ICIs in mRCC.

Finally, DCs with complex immune function phenotypes may also influence the efficacy of ICIs. HLA-DR^hi^ CD83^+^ DC-LAMP^+^ TLS-DC subpopulations, which belong to the immune-activated phenotype, activate CD4^+^ T cells and CD8^+^ TILs by processing and presenting antigens, as well as inducing their clonal proliferation and immune response (38). However, as mentioned above, TLS-DCs were less abundant in ccRCC. In contrast, the CD209^+^ CD83^−^ NTLS-DC subpopulation has an immunosuppressive phenotype and develops under the stimulation of various tumor-derived factors, including IL-10, TGF-β, prostaglandin E2 (PGE2), and chemokines (44). NTLS-DCs, the major DC subpopulation in ccRCC, secrete high levels of MMP-9 and tumor necrosis factor-α, which promote tumor cell growth and invasion (46, 87). NTLS-DCs can selectively promote the proliferation of Tregs in a TGF-β-dependent way, but also inhibit the function of effector CD8^+^ TILs through the L-arginine metabolic pathway (Figure 1D) (88–90). Therefore, we speculate the large ratio of NTLS-DCs to TLS-DCs in ccRCC enhances the robustness of the immunosuppressive microenvironment, which interferes with the activity of ICIs. Finally, it should be noted that the density of a single DC subpopulation may not fully represent the immune status of the tumor microenvironment, as different DC subpopulations may have different immunophenotypes and function in ccRCC.

### The Bidirectional Relationship Between Targeted Therapies and TIME in ccRCC

The effectiveness of targeted therapies is largely dependent on mutations of the drug target and the corresponding signaling pathway. However, the TIME can also influence angiogenesis in ccRCC and eventually lead to resistance to anti-angiogenic agents. In contrast to the cell-autonomous resistance of tumors caused by genomic or epigenetic changes, the non-cell-autonomous resistance caused by the TIME may be more dynamic and complex. Furthermore, besides immunotherapy, targeted therapies (including VEGF/VEGFR inhibitors and mTOR inhibitors) may have immunomodulatory effects on ccRCC and may remodel the TIME. Therefore, understanding the mutual influences between targeted therapies and the TIME is instructive for optimizing the first-line combination therapy or second-line regimens for patients with advanced ccRCC.

### Resistance to VEGF/VEGFR Inhibitors in TIME

**Figure 2:**
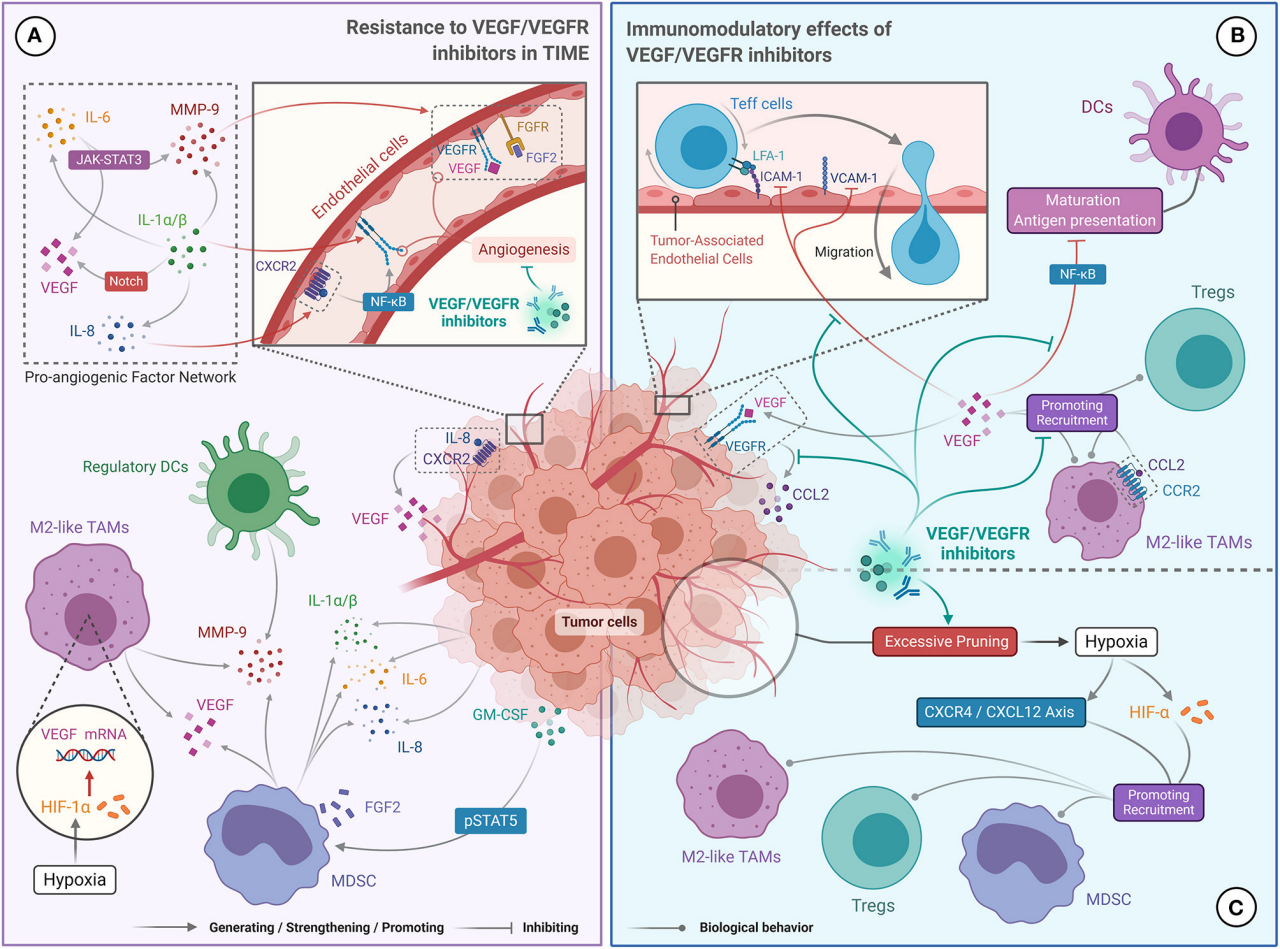
The Bidirectional Relationship between targeted therapies (VEGF/VEGFR inhibitors) and TIME in ccRCC. **(A)** Resistance to VEGF/VEGFR inhibitors is due to a complex network of pro-angiogenic factors (IL-1, IL-6, IL-8, MMP-9, VEGF, and FGF2), which promote excessive tumor angiogenesis. IL-1 promotes the production of IL-6, IL-8, and MMP-9, and enhances the expression of VEGF and VEGFR via the Notch pathway. IL-6 upregulates MMP-9 and VEGF levels via the JAK-STAT3 pathway. IL-8 promotes the secretion of VEGF and the self-activation of the VEGFR. MMP-9 enhances the angiogenic effect of VEDF. Tumor produced GM-CSF can also promote MMP-9 and IL-8 production. Finally, HIF-α promotes VEGF secretion. **(B)** The role of VEGF/VEGFR inhibitors in stimulating the immune response was achieved by blocking the immunosuppressive effect of VEGF. VEGF/VEGFR inhibitors can prevent VEGF-mediated recruitment of TAMs and Tregs, restore DC maturation and antigen presentation, and promote Teff cell migration to tumor microenvironment. **(C)** Immunosuppression mediated by VEGF/VEGFR inhibitors may have resulted from hypoxia. High doses of VEGF/VEGFR inhibitors can excessively prune tumor vessels, leading to hypoxia in the tumor microenvironment, which facilitates recruitment of Tregs, TAMs and MDSCs.

Growing evidence suggests that MDSCs enhance resistance to anti-angiogenesis via a range of non-immune related pathways in multiple tumors, including ccRCC (91). Specifically, MDSCs activate alternative pro-angiogenic pathways by producing multiple pro-angiogenic factors, including VEGF and fibroblast growth factor 2 (FGF2) (Figure 2A) (91, 92). VEGF and FGF2 stimulate the migration and proliferation of tumor-associated endothelial cells, leading to tumor angiogenesis and stability in RCC (93, 94). In addition, granulocyte-macrophage colony-stimulating factor (GM-CSF) secreted by tumor cells mediates the proliferation of MDSCs through the pSTAT5 pathway, and induces them to secrete pro-angiogenic proteins including IL-8 and matrix metalloproteinase-9 (MMP-9), resulting in reduced sensitivity to VEGF/VEGFR inhibitors in RCC (Figure 2A) (95). Besides dynamically remodeling the extracellular matrix, MMP-9 can enhance the pro-angiogenic effect of VEGF (96). Therefore, the presence of MDSCs in the TIME causes RCC to become unresponsive to anti-angiogenic drugs.

M2-like TAMs have also been shown to promote tumor vascularization by producing multiple angiogenic factors. In a hypoxic tumor microenvironment, M2-like TAMs can produce high levels of VEGF-A and hypoxia-inducible factor (HIF)-1α. VEGF-A activates tumor angiogenesis by binding to VEGFR, and HIF-1α enhances this process by upregulating VEGF expression (Figure 2A) (97). In breast and colon cancers, high levels of TAM infiltration were associated with increased expression of proteins related to the Wnt pathway (i.e., Wnt5a and Wnt7b), which is involved in the regulation of angiogenesis (98–100). M2-like TAMs can also secrete MMP-9 to mobilize VEGF (Figure 2A) (101). Indeed, the phase 3 COMPARZ study showed that high infiltration of TAMs was associated with poor prognosis in mRCC patients treated with VEGFR-TKIs, suggesting that TAMs may contribute to the resistance of RCC to anti-angiogenic agents (102).

Cytokines in the tumor microenvironment, which are mainly secreted by MDSCs and tumor cells, are also thought to assist tumor angiogenesis. For example, the pro-inflammatory cytokines IL-1α and IL-1β can induce VEGF and VEGFR2 expression via the Notch pathway, and upregulate MMP-9, IL-6, and IL-8 levels, to form a pro-angiogenic factor network (Figure 2A) (103). Significantly increased levels of IL-1α and IL-1β were observed by Carbone et al. in pancreatic cancers that were resistant to VEGF inhibitors (104). Alternatively, IL-6 can promote the expression of downstream genes, including VEGF and MMP-9, by activating the JAK/STAT3 cascade (Figure 2A) (105, 106). IL-8 interacts with its receptor (CXCR2) to activate NF-κB and promote VEGF expression and VEGFR2 autocrine activation (Figure 2A) (11). Ishibashi et al. confirmed that RCC overexpressed IL-6 and showed drug resistance under VEGFR-TKI treatment, and significant tumor regression was observed after blocking the IL-6 receptor (12). Likewise, Huang et al. demonstrated that IL-8 is an important factor for the resistance of ccRCC to VEGFR-TKI (107). Therefore, the presence of IL-1, IL-6, and IL-8 in the TIME may promote the resistance of ccRCC to VEGF/VEGFR inhibitors by multiple non-redundant pathways.

### Immunomodulatory Effects of VEGF/VEGFR Inhibitors

Numerous preclinical studies have found that VEGF serves a dual function in angiogenesis and immunosuppression. VEGF recruits CD4^+^ Foxp3^+^ Tregs to the tumor microenvironment by interacting with neuropilin 1 on their cell surface (108). Similarly, VEGF promotes the migration of CD11b^+^ MDSCs and TAMs into the tumor microenvironment by binding to the VEGFR on the surface of these cells (Figure 2B) (109, 110). In a ccRCC xenograft model, the binding of VEGF to VEGFR-1 prompted tumor cells to secrete CCL2, which mediated the infiltration of TAMs (111). Besides, binding of VEGF to its receptor inhibits the maturation of DCs and antigen presentation, primarily by blocking the activation of NF-κB (Figure 2B) (110). VEGF also restricts the migration of Teff cells into the tumor microenvironment by downregulating the expression of adhesion molecules including ICAM-1 and VCAM-1 on tumor-associated endothelial cells (Figure 2B) (112).

Overall, the evidence suggests that VEGF recruits immunosuppressive cells to the tumor microenvironment, thereby reducing the anti-tumor immune response. Therefore, VEGF/VEGFR inhibitors may reverse the immunosuppressive nature of the TIME. Indeed, in a clinical trial of bevacizumab combined with atezolizumab for the treatment of mRCC, Wallin et al. found that bevacizumab promoted tumor-specific T-cell infiltration and enhanced the tumor-specific immune response (9). Interestingly, sunitinib has also been shown to improve the anti-tumor response of Teff cells and reduce the recruitment of Tregs and MDSCs (113, 114). Zizzari et al. also found that pazopanib promotes DC activation in mRCC by inhibiting the p-Erk and Wnt-β-catenin pathways (19). Therefore, VEGF/VEGFR inhibitors can inhibit the recruitment of immunosuppressive cells to the tumor microenvironment, restore the function and phenotype of APCs, and promote infiltration of Teff cells, which supports the synergistic effect of immunotherapy and anti-angiogenic therapy. Several phase III clinical trials showed that PD-1/PD-L1 inhibitors combined with anti-angiogenic agents significantly improved survival and therapeutic response in untreated patients with advanced ccRCC compared with anti-angiogenic monotherapy (70, 71, 76). Currently, the latest National Comprehensive Cancer Network (NCCN) and European Society for Medical Oncology (ESMO) guidelines recommend pembrolizumab combined with axitinib as the first-line treatment for mRCC patients from all International Metastatic RCC Database Consortium (IMDC) risk categories (115, 116). Likewise, the combination of VEGF/VEGFR inhibitors with IFN-α had also yielded favorable results. A multicenter phase III trial (AVOREN) of mRCC showed that, when compared with single-agent IFN-α, IFN-α combined with bevacizumab significantly increased PFS (10.2 vs. 5.4 months; hazard ratio [HR] = 0.63, 95% confidence interval [95% CI] 0.52–0.75; *p* = 0.0001) and objective response rates (ORR) (31 vs. 13%; *p* = 0.0001), and did not lead to significantly increasing or new adverse reactions (117). Therefore, the combination of IFN-α and bevacizumab is currently recommended by the European Society for Medical Oncology (ESMO) guidelines as a first-line option for mRCC patients with favorable risk (category 3B) or intermediate risk (category 2C) (116).

Despite this, VEGF/VEGFR inhibitors may also have immunosuppressive effects in some cases. For example, increased infiltration of CD4^+^ Foxp3^+^ Tregs and upregulation of PD-L1 expression were observed in primary RCC patients treated with sunitinib (118). Several studies have also shown that high doses of anti-angiogenic agents could lead to hypoxia of the tumor microenvironment and upregulation of the CXCR4/CXCL12 axis and HIF-α levels due to excessive pruning of tumor vessels, which facilitates the recruitment of TAMs, MDSCs, and Tregs (Figure 2C) (119, 120).

Based on these observations, we propose the following conjecture: moderate doses of VEGF/VEGFR inhibitors are beneficial for enhancing anti-tumor immune responses, while excessive doses can cause hypoxia-induced immunosuppression, which could partially explain the development of acquired resistance and progression in some mRCC patients treated with anti-angiogenic agents alone. Therefore, the dual modulatory effects of anti-angiogenic drugs on the TIME should be considered when choosing the individualized treatment in patients with advanced ccRCC. It is also worth exploring how to determine the optimal dose of anti-angiogenic drugs and how to reduce their immunosuppressive effects.

### Immunomodulatory Effects of mTOR Inhibitors

As a downstream effector of the PI3K/Akt pathway, mTOR regulates various modulators of cell growth (e.g., eIF4E, S6K1, and cyclin-D) and pro-angiogenic factors (e.g., HIF, bFGF, and VEGF) (121, 122). Several studies have shown that the levels of mTOR pathway-related proteins (including p70S6K, p-mTOR, PI3K, and pAkt) in RCC were significantly higher than those in normal kidney tissues, and positively correlated with tumor progression (122). mTOR inhibitors can effectively inhibit tumor proliferation and angiogenesis in RCC and are recommended as second-line therapies for patients with mRCC (115). In fact, mTOR inhibitors were first approved for the prevention of immune rejection in solid organ transplant recipients because of their immunosuppressive properties (123). Thus, it is hypothesized that mTOR inhibitors may also have immunomodulatory functions in the tumor microenvironment.

An increased percentage of Tregs and MDSCs, as well as a decreased frequency of CD56^bright^ NK cells and DCs, were found in mRCC patients treated with the mTOR inhibitor everolimus (124). These results suggest that mTOR inhibitors can promote immunosuppression of the tumor microenvironment in RCC, which limits their anti-cancer efficacy. As cyclophosphamide (CTX) was previously shown to selectively suppress Tregs and restore effector function of Teff cells and NK cells (125), a phase I clinical study attempted to assess whether CTX can counteract the immunosuppression of everolimus (126). CTX combined with everolimus significantly reduced the percentage of Tregs and MDSCs and increased the frequency of CD8^+^ T cells and DC subsets in mRCC patients (126). Currently, the efficacy and safety of this combination therapy are being evaluated in a phase II trial. Thus, using treatments that modulate immunosuppressive cells or enhance the immune response may improve the therapeutic effect of mTOR inhibitors in mRCC.

### Genomic Changes in ccRCC that Influence the TIME

ccRCC has relatively unique genomic features compared to other RCC types, namely chromosomal 3p deletion (>90%), chromosomal 5q gain (>67%), and somatic mutations closely related to 3p deletion events, including mutations in *VHL, PBRM1, SETD2*, and *BAP1* (127). Other common genomic alterations in ccRCC include chromosome 14q deletions, *MTOR* mutations, and *PTEN* mutations (31). Analyses of tumor evolutionary trajectories have shown significant intra-tumor heterogeneity in ccRCC (128); that is, the majority of mutations in ccRCC are subclonal, indicating the existence of significant variations in most trunk mutations from different individuals.

In recent years, the correlation between tumor genomic features and the TIME has received increasing attention. A growing number of studies have found that the TIME of ccRCC presents inherent complexity and individual differences under the manipulation of a heterogeneous genomic landscape, which can partly explain the different responses of advanced ccRCC to immunotherapy or combination therapy.

### *VHL* Mutations

**Figure 3:**
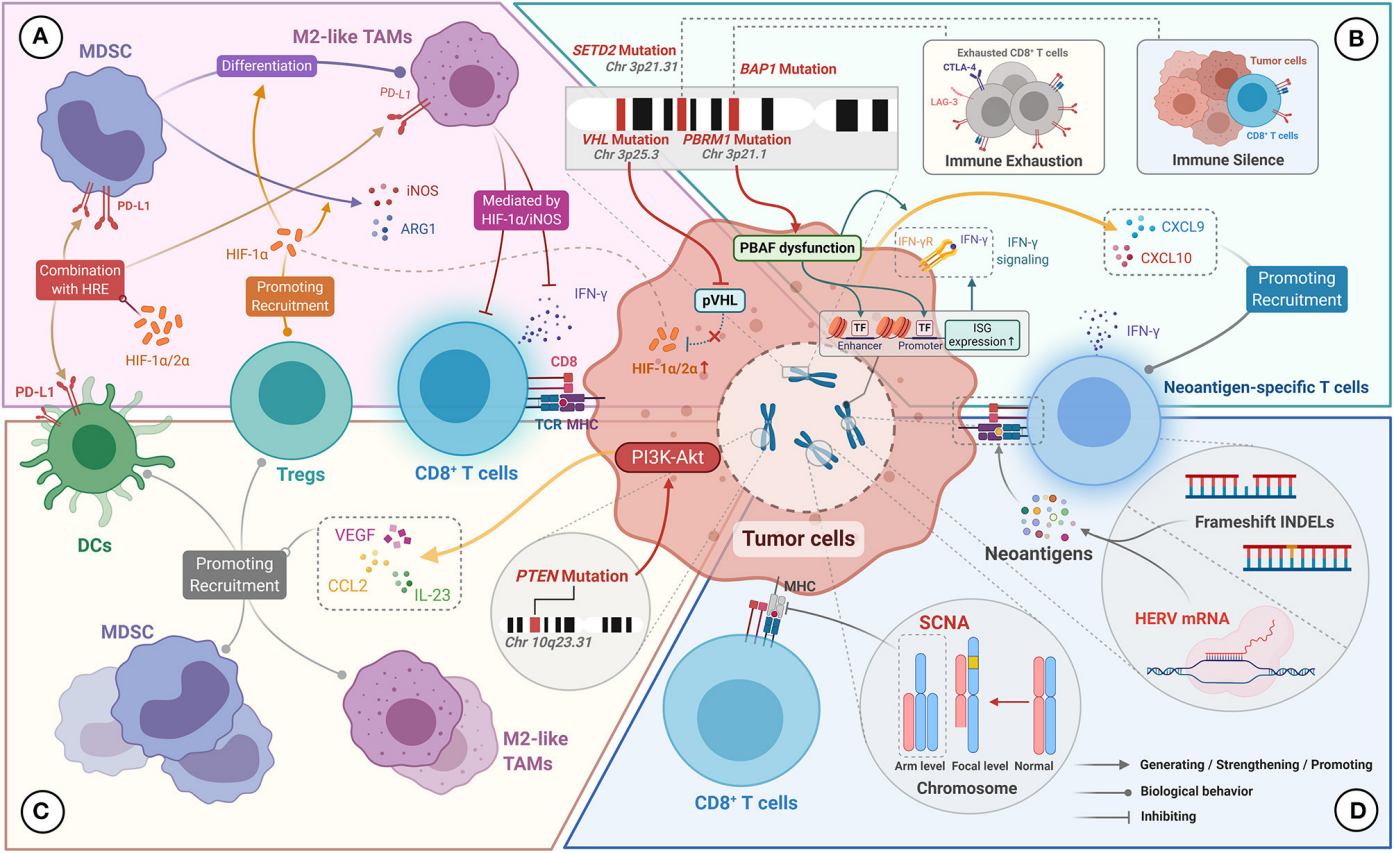
Genomic characteristics of ccRCC for manipulating the TIME. **(A)** *VHL* mutations result in reduced pVHL production and reduced HIF-1α/2α degradation. Excess HIF-1α/2α upregulates the expression of PD-L1 on MDSC, M2-like TAMs, and DCs by binding to the hypoxia response element (HRE). HIF-1α promotes MDSCs to produce iNOS and Arg-1 and to differentiate into M2-like TAMs. HIF-1α promotes the recruitment of Tregs, and mediates the inhibitory effect of M2-like TAMs on the effector function of CD8^+^ TILs. **(B)** *PBRM1* mutation leads to PBAF complex dysfunction, which upregulates interferon-stimulated gene (ISG) expression, thereby enhancing the tumor killing effect mediated by IFN-γ signaling. PBAF inactivation also promotes the secretion of CXCL9 and CXCL10 by tumor cells. *BAP1* mutations are associated with the immuno-exhausted tumor microenvironment. *SETD2* mutations are associated with the immune-silenced tumor microenvironment. **(C)** *PTEN* mutations activate the P13K-Akt pathway, resulting in upregulation of VEGF, CCL2, and IL-23 expression, which contribute to the recruitment of Tregs, DCs, and MDSCs. **(D)** Frameshift INDELs and HERV expression can generate abundant neoantigens, which stimulate the production of neoantigen-specific T cells. Arm level SCNA can disrupt the antigen-presenting capacity of MHC on tumor cells, resulting in inactivation of tumor-specific immune responses.

The *VHL* deletion mutation is located at the short arm of chromosome 3 (3p25.3) and is the most common mutation in ccRCC (found in approximately 80% of cases) (129). The protein encoded by *VHL* has E3 ubiquitin ligase activity and can degrade HIF-α that modulates glucose metabolism and angiogenesis in a hypoxic environment (130). *VHL* deficiency in ccRCC will lead to the accumulation of HIF-α (including HIF-1α and HIF-2α) (Figure 3A), which causes ccRCC to present a unique pathologic manifestation, namely glycogen and lipid accumulation and abundant angiogenesis (127). Besides, under conditions of HIF-1α excess, MDSCs secrete more inducible nitric oxide synthase (iNOS) and Arg-1, and have a greater tendency to differentiate into TAMs with an immunosuppressive phenotype (Figure 3A) (85). iNOS participate in immunosuppression by catalyzing NO formation (131). TAMs preferentially inhibit T-cell proliferation and IFN-γ expression under the action of HIF-1α/iNOS (132). HIF-1α, which stably exists in the tumor microenvironment, could also facilitate the recruitment of Foxp3^+^ Tregs by a TGF-β-independent mechanism (Figure 3A) (133). In addition, using a ccRCC model, HIF-1α and HIF-2α were found to upregulate PD-L1 expression on MDSCs, TAMs, and DCs through directly binding to hypoxia response element (HRE) (Figure 3A) (134, 135). Therefore, overall, *VHL* deletion mutations in ccRCC lead to a large amount of HIF-α accumulation and indirectly promote the formation of immunosuppressive microenvironment via HIF-α-mediated immunosuppressive cell recruitment and upregulation of immunosuppressive molecules.

### *PBRM1, BAP1*, and *SETD2* Mutations

In ccRCC, somatic mutations in *PBRM1* (38.0%), *STED2* (13.2%), and *BAP1* (11.0%) located on the short arm of chromosome 3 are strongly associated with 3p deletion events (31). The bromodomain-containing proteins encoded by the *PBRM1* gene participate in the construction of the PBRM1-Brg1/Brm-associated factors (PBAF) chromatin remodeling complex that is involved in DNA repair processes (136). Meanwhile, histone methyltransferase encoded by the *SETD2* gene is involved in the methylation of histone H3 lysine 36 (H3K36), which plays a role in homologous recombination repair and genome stabilization (137). Indeed, loss of *SETD2* has been shown to cause an increased frequency of deletion-associated mutations (137). The *BAP1* gene encodes the BRCA1 associated protein-1, which influences the cell cycle by regulating the activity of key proteins involved in various cellular processes (138). All three genes are involved in the biological pathways related to tumorigenesis and have frequent mutations, supporting their role as tumor suppressors in ccRCC (139).

The loss of PBAF function caused by *PBRM1* deletion mutations has an impact on the TIME (22). On the one hand, PBAF inactivation enhances the chromatin accessibility of transcription factors on the promoters or enhancers of IFN-γ-inducible genes, leading to increased sensitivity of tumor cells to IFN-γ (Figure 3B). On the other hand, PBAF inactivation promotes the secretion of chemokines CXCL9 and CXCL10 by tumor cells, which contributes to the increased recruitment of Teff cells to the tumor microenvironment (Figure 3B) (22). Furthermore, follow-up results from the CheckMate 025 trial showed that *PBRM1* mutations were strongly associated with improved PFS and OS in advanced ccRCC patients treated with nivolumab, which was not observed in the everolimus group (140, 141). Similar results were reported by Sarah et al. (142). We thus speculate that the *PBRM1* mutation makes tumor cells more sensitive to T-cell-mediated cytotoxicity, and may help improve the therapeutic response to PD-L1/PD-1 blockade therapies. However, no correlation between PBRM1 mutations and OS was observed in a study including 143 patients with metastatic ccRCC treated with ICIs (143). These seemingly conflicting results suggest that the immunostimulatory effects of PBRM1 mutations may be confounded by their direct effects on the biological behavior of ccRCC. Besides, several studies have shown that PBRM1 loss was associated with enhancement of angiogenesis (144, 145), which may affect the response to ICIs of ccRCC by preventing immune cell infiltration. However, it is noteworthy that the above evidence indicated no adverse effects of PBRM1 mutations in patients with advanced RCC treated with ICIs. Overall, further prospective studies to clarify the predictive value of PBRM1 mutations for the therapeutic effects of immunotherapy are warranted given the discrepancy in the results of different studies.

Beuselinck et al. carried out unsupervised clustering analysis for the molecular characteristics of 53 patients with advanced ccRCC and classified them into four four subtypes (ccRCC1 to ccRCC4) (146). The results showed that *SETD2* mutations were related to the immune desert subtype with the poorest T-cell infiltration and lower expression of immunosuppressive markers (ccRCC1). In contrast, *BAP1* mutations were related to the inflammatory subtype with the highest T-cell infiltration (ccRCC4). Among these subtypes, ccRCC4 tumors had the poorest prognosis, which correlated with a high expression of immunosuppressive markers (including PD-L1, PD-1, LAG-3, and TIM-3) and excessive T-cell exhaustion. Besides, ccRCC4 tumors showed no response to sunitinib, which was related to high levels of expression of markers of Tregs (i.e., FOXP3, IL-10., and TGF-β). Similar results were obtained in a study on the immune characterization of ccRCC tumor grafts (147), in which *BAP1* mutations were associated with a highly inflammatory immune phenotype with abundant T-cell infiltration and poor prognosis. Taken together, we speculate that *SETD2* mutations may mediate immune silencing, while *BAP1* mutations may be involved in regulating T-cell infiltration and exhaustion in the TIME in ccRCC (Figure 3B). However, the above conclusions on SETD2 and BAP1 mutations were solely observational in nature, and further investigations were required to determine the immunomodulatory mechanism of them.

### *PTEN* Mutations

The *PTEN* gene is one of the most commonly mutated tumor suppressor genes in human cancer, and negatively regulates the PI3K/AKT pathway by encoding a protein with phosphatase activity (148). *PTEN* mutations, which occur in approximately 4.5% of cases of ccRCC, remove the inhibitory effect on the PI3K/AKT pathway, leading to increased proliferation and migration of tumor cells (31, 148). Several studies have found that the sustained activation of the PI3K/AKT pathway caused by *PTEN* mutation upregulates the secretion of several immunosuppressive cytokines, including VEGF, IL-23, and CCL2 via NF-κB-JAK/STAT3 signaling (Figure 3C) (149–152). In addition to its role in angiogenesis, VEGF can recruit immature DCs, MDSCs, and Tregs to help sustain the immunosuppressive tumor microenvironment (153, 154). Moreover, *PTEN* loss can increase tumor cell resistance to T-cell killing by significantly upregulating CCL2 and VEGF (155). Besides, *PTEN*-deficient metastatic uterine smooth muscle sarcoma, accompanied by increased expression of VEGF-A and STAT3, were resistant to PD-1/PD-L1 therapy (156). More critically, *PTEN* mutations observed in ccRCC correlated with high infiltration of M2-like TAMs (157). Overall, *PTEN* mutations may upregulate the expression of multiple immunosuppressive factors by eliminating the negative regulation of the downstream PI3K/AKT pathway, thus inducing the immune escape of tumor cells.

### Human Endogenous Retroviruses

HERVs form about 8% of the human genome, and are predominantly located in heterochromatin (158). Throughout millions of years of evolution, exogenous retroviruses have repeatedly infected hosts and integrated into their genomes, leading to the formation of HERVs. Under normal physiological conditions, most HERVs are usually inactive because of the presence of epigenetic silencing. However, HERV expression can be induced due to the lack of CpG methylation in tumor tissues (159). In a pan-cancer analysis of HERVs, Smith et al. found that HERV-derived proteins could participate in the regulation of the TIME and correlated with prognosis, particularly in ccRCC (160). Significant upregulation of HERV expression was observed in ccRCC that were responsive to PD-1 blockade, and positively correlated with Teff cell infiltration and the level of cytotoxic markers. Further studies revealed that HERV expression products could serve as tumor-specific antigenic epitopes to induce HERV-specific T cell production (Figure 3D) (160). Taken together, HERVs could be a source of neoantigens and contribute to the cytotoxic effects of Teff cells, thereby shaping the TIME.

### Tumor Mutational Burden and Somatic Copy-Number Alterations

The observation that patients with higher TMB are more likely to respond to PD-1/PD-L1 blockade has been reported in a variety of malignant tumors (161). High TMB indicates the presence of more mutation-associated antigens (MANA) in the tumor microenvironment, which facilitates the induction of MANA-specific T cells and activation of the adaptive anti-tumor immune response (162). However, it has been found that ccRCC has a lower TMB than other malignancies that were responsive to immunotherapy, which seems inconsistent with the above point (163).

The TMB mainly depends on the reads of non-synonymous single nucleotide variation (nsSNV) in the tumor cell genome. Frameshift mutations caused by insertional or deletion mutations (INDELs) can similarly produce immunogenic neoantigens (Figure 3D) (164). In a pan-cancer analysis, ccRCC had the highest frequency of frameshift INDELs, which seems to explain the phenomenon that PD-1/PD-L1 inhibitors are effective for the treatment of ccRCC with low TMB. However, in the genomic analysis of the IMmotion150 trial, no significant association was found between TMB or frameshift mutation burden (FMB) and the response to atezolizumab alone (anti-PD-L1) or in combination with bevacizumab (anti-VEGF) (14). Similarly, there was no significant correlation between TMB or FMB and the gene signature of Teff cells in the TIME.

In tumor genome aneuploidy studies, Davoli et al. showed that the expression levels of genes associated with adaptive immunity and the ratio of pro-inflammatory cytokines to immunosuppressive molecules were significantly reduced in tumors with high levels of arm-level SCNA (including ccRCC) (165). This finding indicates that tumors with high levels of SCNA may have more significant immunosuppressive properties. The authors hypothesized that arm-level SCNA could impair the antigen-extraction capacity of MHC or affect the balance of proteins associated with cytotoxic immune cell infiltration, resulting in blocking the activation of the tumor-specific immune response (Figure 3D) (165). Overall, frameshift INDELs and arm-level SCNA in ccRCC appear to exert diametrically opposed effects on the TIME, and thus, their underlying mechanisms should be further explored.

### Novel Therapeutic Strategies for Advanced ccRCC Based on the TIME

As emphasized above, various immune cell subsets and immunomodulatory molecules in the TIME impact on the response to immunotherapy and targeted therapy. Moreover, with the in-depth study of the TIME, multiple immune regulation pathways have been found to affect tumor cell survival. Therefore, by targeting different immune microenvironment components or non-redundant immunomodulatory pathways, we may be able to overcome the therapeutic resistance of advanced ccRCC.

### Other Immune Checkpoint Molecules

Besides PD-1 and CTLA-4, other immune checkpoints (e.g., LAG-3 and Tim-3) are involved in the immune escape of tumor cells and resistance to ICIs. Therefore, strategies targeting these other immune checkpoints have great therapeutic potential. Indeed, several early clinical trials of anti-LAG-3 monoclonal antibodies or anti-Tim-3 monoclonal antibodies combined with PD-1 inhibitors are being conducted in several tumor types, including RCC (Table 1). Besides, a bispecific antibody (bsAb) that combines two two immune checkpoints is receiving increasing attention, which can reactivate Teff cells more efficiently by blocking different immunosuppressive pathways. Currently, XmAb22841 (a bsAb targeting CTLA-4 and LAG-3) is being evaluated in a phase I trial in mRCC patients (Table 1). Stimulatory checkpoint molecules, including OX40 (also known as CD134) and CD27, are also potential therapeutic targets for the treatment of tumors such as RCC. OX40, mainly expressed on activated T cells, stimulates T cell proliferation and enhances effector function when binding to its ligand (OX40L) expressed on antigen-presenting cells (166). CD27, mainly expressed on T cells and NK cells, enhances NK cell-dependent and T cell-dependent anti-tumor immunity when binding to CD70 (166). Thus, activating co-stimulatory molecules can enhance the anti-tumor immune response by a mechanism distinct from inhibitory checkpoint molecules, which is a novel immunotherapeutic strategy for mRCC (167). Indeed, PF-04518600 (an OX40 agonist) combined with axitinib and varlilumab (a CD27 agonist) combined with nivolumab are currently in clinical development for mRCC (Table 1).

**Table 1:** Novel treatment strategies for advanced ccRCC based on the tumor immune microenvironment.

**Drug Type**	**NCT Number**	**Intervention**	**Comparison**	**Histology**	**Primary endpoint**	**Status**	**Phase**	**Number of patients**	**Allocation**
Immune checkpoint molecules	NCT01968109	Relatlimab (anti-LAG-3) +/– Nivolumab	–	RCC and others	Safety, efficacy	Recruiting	Phase I/II	1,500	Randomized
	NCT02996110	Relatlimab + Nivolumab	Nivolumab + Ipilimumab	RCC	Safety	Recruiting	Phase II	200	Randomized
	NCT02460224	LAG525 (anti-LAG-3) +/– PDR001 (anti-PD-1)	–	RCC	Safety, ORR	Active, not recruiting	Phase I/II	490	Non-randomized
	NCT02608268	MBG453 (anti-Tim-3) +/– PDR001 (anti-PD-1)	–	RCC	Safety, ORR	Recruiting	Phase I/II	269	Non-randomized
	NCT03849469	XmAb22841 (anti-CTLA-4 & LAG-3) +/–Pembrolizumab	–	RCC	Safety	Recruiting	Phase I	242	Non-randomized
	NCT03092856	PF-04518600 (OX40 agonist) + Axitinib	Axitinib	ccRCC, nccRCC	PFS	Recruiting	Phase II	104	Randomized
	NCT02335918	Varlilumab (CD27 agonist) +Nivolumab	–	RCC and others	ORR	Completed	Phase II	175	N/A
Modified cytokines	NCT03729245	NKTR-214 (CD122 agonist) + Nivolumab	Sunitinib or Cabozantinib	RCC	OS, ORR	Recruiting	Phase III	600	Randomized
	NCT02009449	AM0010 (PEG-IL-10)	–	RCC and others	Safety	Active, not recruiting	Phase I	350	Non-randomized
Small-molecule immunomodulators	NCT02667886	X4P-001 (CXCR4 inhibitor) +/– Axitinib	–	ccRCC	Safety	Active, not recruiting	Phase I/II	74	Randomized
	NCT02923531	X4P−001 + Nivolumab	–	ccRCC	Safety	Completed	Phase I/II	9	N/A
	NCT02675439	MIW815 (STING agonist) +/– Ipilimumab	–	RCC and others	Safety, RD	Active, not recruiting	Phase I	47	Non-randomized
Targeting immunometabolism	NCT02655822	Ciforadenant (A2aR antagonist) +/– Atezolizumab	–	RCC and others	Safety, ORR, DLT, MDL	Recruiting	Phase I	336	Randomized
	NCT02754141	BMS-986179 (anti-CD73) +/– Nivolumab or rHuPH20	–	RCC and others	Safety	Recruiting	Phase I/II	268	Non-randomized

*A2aR, adenosine A2a Receptor; ccRCC, clear cell renal cell carcinoma; CTLA-4, cytotoxic T-lymphocyte-associated protein 4; DLT, dose-limiting toxicities; LAG-3, lymphocyte-activation gene 3; MDL, maximum dose level; nccRCC, non-clear cell renal cell carcinoma; ORR, objective response rate; OS, overall survival; PD-1, programmed cell death-1; PEG-IL-10, pegylated interleukin-10; PFS, progression-free survival; RCC, renal cell carcinoma; RD, Recommended dose; rHuPH20, recombinant human hyaluronidase PH20 enzyme; STING, stimulator of interferon genes; Tim-3, T-cell immunoglobulin mucin-3*.

### Modified Cytokine Therapies

NKTR-214 (also called bempegaldesleukin) is a PEGylated IL-2 that drives proliferation and activation of CD8^+^ TILs and NK cells within the TIME by binding to the dimeric IL2Rβγ (CD122). Compared to traditional IL-2, NKTR-214 does not cause significant amplification of CD4^+^ Foxp3^+^ Tregs, and thus, has greater anti-tumor activity and fewer adverse effects (168). This is because NKTR-214 has a limited affinity for IL2Rα subunit, and thus, cannot bind to the IL2Rα*βγ* heterotrimer on Tregs (169). Currently, NTRK-214 combined with nivolumab is being evaluated in mRCC in the phase III PIVOT-09 trial (Table 1). AM0010 (also called pegilodecakin) is a modified PEGylated IL-10 that may enhance the anti-tumor immune response by harnessing the immunostimulatory function of IL-10. The latest results from the ongoing phase I/Ib IVY study confirm that second-line treatment with AM0010 in combination with a PD-1 inhibitor has a good response rate in mRCC (ORR: 33–43%), which is even better than the currently recommended second-line regimen (Table 1) (170).

### Small-Molecule Immunomodulators

Activation of the CXCR4/CXCL12 pathway is associated with the formation of an immunosuppressive tumor microenvironment (171), and X4P-001 (a CXCR4 antagonist) can block excessive activation of this pathway to reverse tumor immune escape (172). Preclinical studies have demonstrated that CXCR4 antagonists could reduce the inhibitory effect of immunosuppressive cells on Teff cells and increase T-cell sensitivity to tumor antigens (173, 174). Currently, combination of X4P-001 with other anticancer therapies are in development (Table 1). Another potential target is the stimulator of interferon genes (STING), which is activated upon binding to cyclic dinucleotide (CDN), and initiates Teff cell-mediated adaptive immunity by inducing type I IFN production and DC activation (175). The STING agonist MIW815 (also called ADU-S100) was found to activate an anti-tumor immune response in preclinical trials (176), and phase I trials of MIW815 alone or in combination with ipilimumab are currently evaluating the efficacy and safety in multiple cancer types, including RCC (Table 1).

### Targeting Immunometabolism

Hypoxia, the rapid proliferation of tumor cells, and upregulated expression of CD39 and CD73 can accelerate adenosine production in the tumor microenvironment (177). Several studies have shown that adenosine inhibits the proliferation and effector function of Teff cells, as well as the maturation and antigen-presenting ability of DCs by binding to A2a receptors (A2AR) (177, 178). Adenosine also upregulates the expression of FOXP3 and immune checkpoints, including PD-1, CTLA-4, and LAG-3 (178). CPI-444 (also called ciforadenant) is a selective inhibitor of the A2AR that reverses the immunosuppressive effect by blocking adenosine signaling. Early results from an ongoing phase I/Ib clinical study showed disease control rate of 75% and 100% for CPI-444 alone and in combination with atezolizumab in mRCC, respectively (Table 1) (179). Additionally, an anti-CD73 antibody (BMS-986179) that targets the pathway toward adenosine production is currently being evaluated in a phase 1 clinical trial (Table 1) (177).

## Conclusion and Future Perspectives

Although ICI-based combination therapies have improved the prognosis of patients with advanced ccRCC, some patients show no response or progress during the treatment process. Increasing evidence suggests that the TIME is an important factor affecting therapeutic response in such cases. Both genomic characteristics and immunomodulatory effects of systemic therapy cause dynamic changes in the TIME in advanced ccRCC, which, in turn, impacts the therapeutic response. Several novel therapeutic strategies optimized according to the components of the TIME are under development to improve outcomes for patients with advanced ccRCC. However, the integration of tumor genomic and immune signatures to more accurately predict therapeutic response is an important task to be refined in the future. Moreover, there are obvious unmet needs in developing the optimal treatment sequencing and combination strategy based on the interaction between the TIME and systemic therapy. We believe that comprehensive correlation analysis combining the TIME, tumor genome, and therapeutic modalities could provide more accurate prediction and decision-making for the individualized treatment of advanced ccRCC patients in the near future.

## Author Contributions

EL, XL, YL, and YY conceived the idea for the research and designed the review. EL and XL created the figures. All authors participated in the drafting and revision of the manuscript, performed the literature review and approved the submitted version.

## Conflict of Interest

The authors declare that the research was conducted in the absence of any commercial or financial relationships that could be construed as a potential conflict of interest.
